# Tolvaptan treatment in an adult Fontan patient with protein-losing enteropathy: a serial ^23^Na-MRI investigation

**DOI:** 10.1177/20406223211004005

**Published:** 2021-04-16

**Authors:** Julia Moosmann, Okan Toka, Peter Linz, Anke Dahlmann, Armin M. Nagel, Mario Schiffer, Michael Uder, Robert Cesnjevar, Sven Dittrich, Christoph Kopp

**Affiliations:** Department of Paediatric Cardiology, Friedrich-Alexander University of Erlangen-Nürnberg, Loschgestraße 15, Erlangen, 91054, Germany; Paediatric and Adolescent Practice, Fürth, Germany; Department of Radiology, Friedrich-Alexander University of Erlangen-Nürnberg, Erlangen, Bayern, Erlangen, Germany/Department of Nephrology and Hypertension, Friedrich-Alexander University of Erlangen, Erlangen, Germany; Department of Nephrology and Hypertension, Friedrich-Alexander University of Erlangen, Erlangen, Germany; Department of Radiology, Friedrich-Alexander University of Erlangen-Nürnberg, Erlangen, Germany; Department of Nephrology and Hypertension, Friedrich-Alexander University of Erlangen, Erlangen, Germany; Department of Radiology, Friedrich-Alexander University of Erlangen-Nürnberg, Erlangen, Germany; Department of Paediatric Cardiac Surgery, Friedrich-Alexander University of Erlangen, Erlangen, Germany; Department of Paediatric Cardiology, Friedrich-Alexander University of Erlangen-Nürnberg, Germany; Department of Nephrology and Hypertension, Friedrich-Alexander University of Erlangen, Erlangen, Germany

**Keywords:** Fontan, Tolvaptan, ^23^Na-MRI, RAAS, Congenital heart disease

## Abstract

**Background:**

Protein-losing enteropathy (PLE) is a severe complication of the univentricular Fontan circulation and associated with disturbances in salt and water homeostasis. Fontan patients with PLE have a poor prognosis, with increased morbidity and mortality. Due to limited therapeutic strategies, patients are often treated only symptomatically.

**Methods:**

We report our first experience of Tolvaptan (TLV) treatment in a Fontan patient with PLE, severe volume retention and hyponatraemia, refractory to conventional diuretic therapy. In addition to clinical parameters, we monitored drug effects including tissue sodium and volume status *via* serial ^23^Na-magnetic resonance imaging (^23^Na-MRI) and bioimpedance spectroscopy compared with age-matched controls.

**Results:**

^23^Na-MRI identified elevated tissue sodium, which decreased under TLV treatment, as well as volume status, while serum sodium increased and the patient’s symptoms improved. During long-term treatment, we were able to differentiate between sodium and volume status in our patient, suggesting that TLV uncoupled body sodium from water.

**Conclusion:**

TLV in addition to loop diuretics improved clinical symptoms of PLE and lowered tissue sodium overload. Long-term effects should be further evaluated in Fontan patients.

## Introduction

The Fontan procedure is a palliative procedure for patients with congenital single-ventricle malformations, diverting blood from the great veins to the pulmonary arteries, bypassing the right ventricle.^1^ The Fontan circulation results in a non-pulsatile pulmonary blood flow, elevated central venous pressure and reduced cardiac output.^2–4^ After the Fontan procedure ~3–15% of all patients develop protein-losing enteropathy (PLE), which is still associated with increased morbidity and an estimated 5-year survival rate of ~50%.^5–7^ PLE in Fontan patients represents a severe complication with a gradual onset of symptoms including hypoalbuminaemia, hypogammaglobulinaemia, diarrhoea, dystrophy, hyponatraemia and fluid overload with pleural effusions, ascites and peripheral oedema.^8^ We do not sufficiently understand the pathophysiological changes leading to PLE in Fontan patients, nor do we have adequate treatment options to improve their state of health and life quality. Patients are usually treated symptomatically with diuretics and steroids; however, the response often remains suboptimal and unsatisfactory. In addition, there are no prospective or controlled data evaluating the impact of chronic diuretic therapy on morbidity and mortality in Fontan patients.

We reasoned that Tolvaptan (TLV), a vasopressin type-2 receptor antagonist, might be a useful adjunctive therapy for these patients. Studies in heart failure patients support this viewpoint. TLV prohibits the movement of aquaporin 2 into the luminal wall of the collecting duct, thus reducing the reabsorption of water. TLV has been approved for the treatment of hyponatraemia associated with congestive heart failure. Studies illustrated the efficacy of TLV in heart failure with hypervolaemia and hyponatraemia, especially during the acute phase of cardiac decompensation and in patients resistant to conventional diuretic therapy.^9,10^ To monitor the drug effects, we employed ^23^Na-magnetic resonance imaging (MRI) – a technique that is applied in biomedical research applications to quantify tissue sodium (Na^+^) concentration.^11,12^ We showed earlier that this method is useful in patients with hypertension and heart failure by delineating increased tissue Na^+^ content.^13,14^

### Case presentation

We present a 22-year-old woman with Fontan circulation and PLE with an underlying cardiac malformation of pulmonary atresia and an intact ventricular septum with a hypoplastic right ventricle. Her surgical history included Blalock-Taussig-shunt (BT) at the age of 10 days, which was converted to a central aortopulmonary-shunt (AP-shunt) at 17 months, requiring banding of the AP-shunt 8 days later, due to overshunting. This was followed by patch enlargement of the left pulmonary artery (LPA) at the age of 2.5 years, and late bidirectional Glenn anastomosis at 9 years. She underwent total-cavopulmonary-connection (TCPC) with an extra-cardiac conduit at 11 years. After Fontan completion, she required interventional stent implantation in the LPA; 3 years after TCPC she developed symptoms of PLE. Due to worsening of her clinical condition, she has been hospitalised since the age of 21 for recurring pleural effusions, need for intravenous diuretics and parenteral nutrition.

PLE was diagnosed using the criteria of the scientific statement of the American Heart Association, including elevated faecal alpha-1 antitrypsin, serum hypoalbuminaemia and symptoms of oedema without another identified cause.^15^

Patients PLE symptoms included severe diarrhoea, hypoalbuminaemia, repeated elevated values of faecal alpha-1 antitrypsin (>1800 µg/g). She presented with peripheral oedema, recurrent ascites, pleural effusion and abdominal distension, as well as cachexia and growth failure. The patient had received oral budesonide treatment for symptom control for years since the first symptoms of PLE occurred. She developed severe side effects of steroid therapy, including osteoporosis with chronic base and top plate fractures of the spine. Therefore, budesonide therapy had been weaned. She showed severe immune abnormalities with hypogammaglobulinaemia, lymphopaenia and low T cell count. In the MRI she presented with thoracic lymphatic malformations. Allupurinol treatment was needed for high uric acid >8.5 mg/dl and gout symptoms (Table 1).

**Table 1.** ^23^Na-MRI, laboratory analyses, TLV dosing and concomitant medication.

	First measurement	Second measurement	Third measurement
Time according to TLV start (days)	(−26)	9	29
Body weight (kg)	52.5	51.0	52.4
Blood pressure (mmHg) Systolic/diastolic/mean	106/44/67	93/42/58	95/42/60
TLV dose (mg)	0	18.75	45
^23^Na-MRI			
Muscle sodium (mmol/l)	24.7	22.4	21.7
Skin sodium (mmol/l)	23.8	18.4	17.4
BCM overhydration (l)	1.0	0.1	0.5
BCM extracellular water (l)	11.5	10.6	10.8
BCM intracellular water (l)	12.6	12.7	12.2
Laboratory parameters			
Serum-Na^+^ (mmol/l)	133	131	133
Serum-K^+^ (mmol/l)	3.6	3.5	3.3
Serum-osmolality (mosm/kg)	299	296	292
Creatinine (mg/dl)	0.61	0.67	0.8
Total-protein (g/l)	39	46	44
Albumin (g/l)	21.2	27.6	22.5
IgG (g/l)	1.3	1.9	2.3
Aspartate-aminotransferase (U/l)	24	29	28
Alanine-aminotransferase (U/l)	23	28	23
Gamma-glutamyltransferase (U/l)	169	171	116
Renin (pg/ml)	9780	8350	9250
Aldosterone (pg/ml)	677.9	540.2	91.2
Urine-sodium (mol/mol/Kre)	18.1	<detection limit	33.8
Fractional sodium excretion (%)	0.74	<detection limit	1.8
Urine-potassium (mol/mol/Kre)	36.4	51.8	80.1
Fractional potassium excretion (%)	54.53	104.63	171.67
Urine-osmolality (mosm/kg)	311	254	236
Concomitant medication			
Furosemide (mg/kg/day i.v.)	5	4	4
Hydrochlorothiazide (mg/kg/day)	1	1	1
Eplerenone (mg/kg/day)	1	1	1
Sildenafil (mg/kg/day)	0.6	0.6	0.6
Levothyroxine (µg/kg/day)	2	2	2
Pantoprazole (mg/kg/day)	1.2	1.2	1.2
Iodide (mg/kg/day)	6	6	6
Methyldigoxin (mg/kg/day)	0.002	0.002	0.002
Losartan (mg/kg/day)	0.188	0.188	0.188
Vitamin D (IU/day)	1000	1000	1000
Allopurinol (mg/kg/day)	2	2	2
Heparin (IU/kg/h PTT 60–80 s)	40	40	40
Cardiac catheterisation (pressure values mmHg)			
Inferior vena cava		9/10/10	
Superior vena cava		10/8/8	
Left pulmonary artery		12/10/8	
Right pulmonary artery		12/9/11	
Ascending aorta		82/39/53	
Cardiac MRI			
EDV (ml/m^2^)		111	
ESV (ml/m^2^)		48	
Stroke volume (ml/m^2^)		63	
Ejection fraction (%)		57	
Aortic insufficiency (%)		5	
Mitral insufficiency (%)		11	

BCM, body composition measurement; EDV, end-diastolic volume; ESV, end-systolic volume; IgG, immunoglobulin G; IU, international units; ^23^Na-MRI, ^23^Na-magnetic resonance imaging; PTT, partial thromboplastin time; TLV, Tolvaptan.

Cardiac catheterisation revealed normal pressures in the Fontan circulation (Table 1). TLV (Samsca®, Otsuka Pharma GmbH, Frankfurt am Main, Germany) therapy was started as an individual healing attempt based on the patient’s volume overload and insufficient response to high-dose conventional diuretic therapy. TLV was started with 25% of the target dose (1.0 mg/kg/day). Increments were based on serum sodium concentration. The target dose was achieved on day 25 of treatment.

### Control group

We recruited an age-matched female control group at the local university (*n* = 8). Average age was 24.6 ± 2.0 years. Subjects had no history of chronic diseases, did not take any regular medication and blood pressure was in the normal range according to European Society of Cardiology/European Society of Hypertension (ESC/ESH) guidelines.

### ^23^Na-MRI quantification

^23^Na-MRI was performed before TLV treatment, on day 9 (short-term effects), and day 29 (long-term effects). Tissue Na^+^ content was measured noninvasively with a 3.0 Tesla clinical MRI system (Magnetom Verio, Siemens Healthcare, Erlangen, Germany) using a ^23^Na volume coil (Stark-Contrast, Erlangen, Germany) as described previously.^13,16^ Muscle and skin sodium were assessed in the left lower leg, which was placed on a calibration tube holder to avoid deviation in the Z-axis. Four tubes containing aqueous solutions with 10, 20, 30 and 40 mmol/l NaCl, respectively, served as calibration standards by relating MR-signal intensity to a sodium concentration in a linear trend analysis. To distinguish the anatomical structures of interest, ^1^H-MRI was conducted with the integrated body coil of the MRI system. Due to the low in-plane resolution (3 × 3 mm^2^), partial volume effects occur, meaning the Na^+^ skin amount could have been underestimated. Additionally, ^23^Na-MRI shows fast signal decay, which also can lead to underestimated tissue Na^+^ content measurements.

### Body composition measurements

A body composition monitor device was used (BCM, Fresenius, Medical Care, Bad Homburg, Germany) to determine volume status. Electrodes were attached to the patient’s hand and foot on the ipsilateral side, and impedance spectroscopy was measured with frequencies ranging from 5 kHz to 1 MHz. While high frequencies pass through the whole body’s water, very low frequencies cannot penetrate cell membranes and thus only pass through the extracellular water (ECW) space. The generated impedance data are applied to calculate total body water (TBW), intracellular water (ICW) and ECW.^17^

## Results

### Clinical and laboratory parameters

TLV treatment reduced the patient’s body weight from 52.5 to a minimum on day 5 (48.9 kg). Pleural effusions, ascites, and peripheral oedema regressed. Clinical well-being improved substantially (improved activity level, less abdominal pain, reduced shortness of breath and improved appetite). We were able to reduce the concomitant diuretic therapy on day 12 of TLV treatment (Table 1). As main side effect, the patient described increased thirst and intermittent headache.

Laboratory values including electrolytes, liver and kidney function and urine analysis at each visit (Table 1). Before commencement of TLV treatment, the patient showed a decreased serum sodium concentration of 129 mmol/l, which increased during treatment to a maximum of 135 mmol/l on days 4 and 5 and remained between 130 and 134 mmol/l during the whole treatment period. At the time of ^23^Na-MRI measurements, 26 days before, at days 9 and 29 of TVL treatment, only slight differences in serum sodium could be detected (Table 1). Plasma renin activity and aldosterone were both elevated before treatment, aldosterone decreased to normal values, while plasma renin activity remained steady. Liver enzymes did not increase during treatment (Table 1).

### Tissue sodium and fluid status

**Figure 1.**
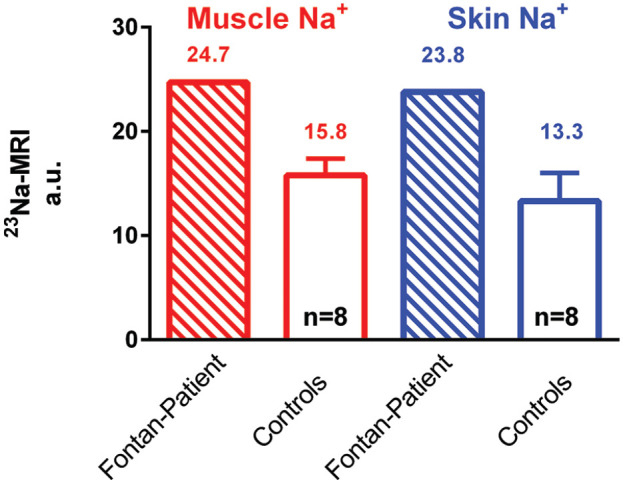
Absolute tissue sodium content of the Fontan patient compared with age/gender-matched healthy controls. Skin and muscle tissue of the Fontan patient before TLV treatment revealed a significant sodium overload. a.u., arbitrary units; ^23^Na-MRI, ^23^Na-magnetic resonance imaging; TLV, Tolvaptan.

**Figure 2.**
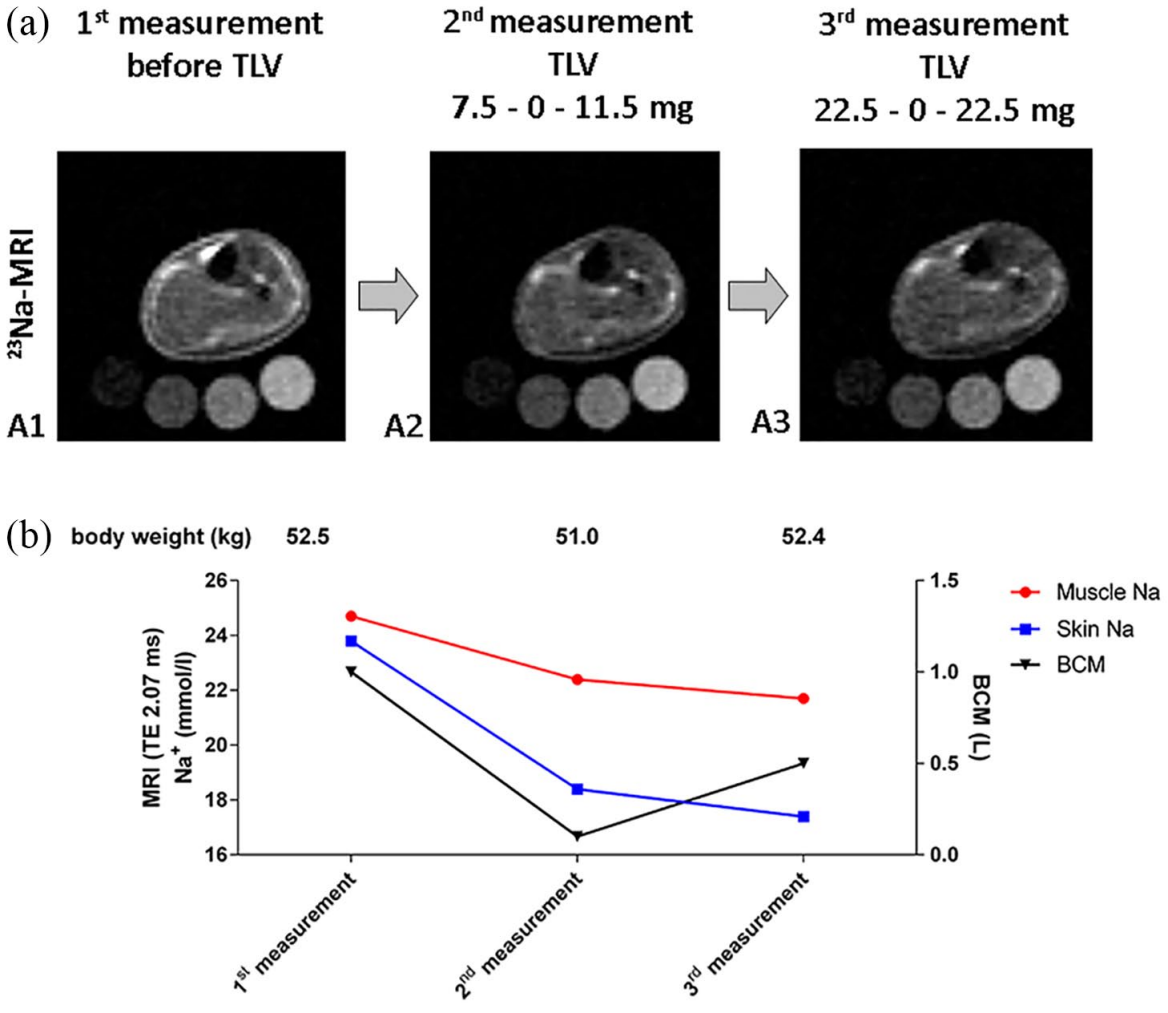
^23^Na-MRI of tissue sodium. (A) ^23^Na-MRI of the left lower leg for assessment of muscle and skin Na^+^: before therapy (A1); and during short-term (A2, day 9) and long-term (A3, day 29) therapy. Four tubes containing 10, 20, 30 and 40 mmol/l of NaCl-standard solution were placed below the lower leg. (B) Anatomic localiser. Graph representing absolute values of tissue Na^+^ (red, muscle; blue, skin), BCM overhydration (black) and body weight. BCM, body composition measurement; ^23^Na-MRI, ^23^Na-magnetic resonance imaging; TLV, Tolvaptan.

The initial image before therapy (−26 days) revealed an increased tissue sodium content in skin (23.8 mmol/l) and muscle (24.7 mmol/l) compared with age-matched female controls (skin 13.3 ± 2.7, muscle 15.8 ± 1.6 mmol/l, *n* = 8, age 24.6 ± 2.1; Figure 1). The second ^23^Na-MRI on day 9, revealed reduced muscle (22.4 mmol/l; reduction of 9.3%) and skin sodium content (18.4 mmol/l; reduction of 22.7%) (Figure 2A, A2). The third ^23^Na-MRI on day 29 of treatment revealed the long-term effect on the target TVL dose of 1 mg/kg/day. ^23^Na-MRI assessment showed a further decrease in tissue sodium in muscle (21.7 mmol/l) and skin (17.4 mmol/l) (Figure 2A, A3). A total reduction of 12.2% in muscle sodium and 26.9% in skin sodium was detected overall after initiating TLV treatment. Despite a further reduction in tissue sodium in muscle and skin, the extracellular water (overhydration) as measured by BCM and total body weight (52.4 kg) increased slightly in our long-term assessment (Figure 2A, A3).

## Discussion

This is the first report of additional TLV treatment in a Fontan patient with PLE and severe, persistent fluid retention where conventional diuretic treatment had proven to be insufficient. The main finding of this serial ^23^Na-MRI investigation was a pronounced accumulation of sodium in muscle and skin, which was mobilised by TLV treatment. While the normalised tissue sodium amount persisted, TLV reduced body water only transiently, illustrating an uncoupling of salt and water homeostasis.

Managing Fontan patients with severe symptoms of PLE remains challenging, and pharmacological strategies are often only symptomatic with limited efficacy over time. TLV has been proposed as a new treatment alternative in biventricular patients with heart failure involving hypervolaemia and hyponatraemia resistant to conventional diuretic therapy.^18^ This motivated us to consider TLV as a new therapeutic agent for our Fontan patient with PLE. The intestinal protein loss in Fontan patients results in reduced albumin and total protein and, therefore, reduced oncotic pressure, hyponatraemia and volume overload. In addition, a chronic inflammatory response is thought to be involved in the development and maintenance of PLE symptoms, affecting cell permeability and contributing to protein loss and oedema.^19^

Although the EVEREST study (Efficacy of Vasopressin Antagonism in Heart Failure Outcome Study with TLV) failed to demonstrate a survival benefit from TLV treatment in patients suffering from exacerbated chronic heart failure, a sub-analysis showed reduced long-term morbidity and mortality for patients with hyponatraemia.^18,20^ In our patient, who had low sodium level before therapy, TLV alleviated pleural effusions and ascites and improved her clinical state of health.

In addition to the clinical improvements, ^23^Na-MRI enabled us to visualise sodium accumulation in muscle and skin during treatment. Before TLV treatment, we identified markedly increased tissue sodium levels. Similar findings have been reported in patients with heart failure.^13^ Despite serum hyponatraemia, Fontan patients seem to be sodium overloaded. ^23^Na-MRI identified a gradually decrease in tissue sodium during TLV treatment, which we did not expect, as TLV is supposed to only increase urinary water excretion. However, the anticipated mobilisation of body water was merely transient and not accompanied by a commensurate change in tissue sodium during long-term treatment. The serum sodium analysis in our patient could not have predicted these changes at the tissue level.

We can only speculate how TLV affects overall tissue sodium-water homeostasis in Fontan patients. A direct effect is excluded since aquaporin-2 channels do not exist in skin and muscle. However, the Vasopressin-2 receptor (V_2_R) is expressed not only in the collecting duct, where it promotes water reabsorption, but can also be found in the ascending limp of the Henle’s loop (TAL) in the kidney. Furthermore, antidiuretic hormone (ADH) – the ligand of V_2_R – is known to increase NaCl reabsorption *via* the NaK2Cl-cotransporter (NKCC2) and additionally by paracellular mechanisms in the TAL.^21,22^ According to these data, one could assume that TLV increases renal NaCl excretion. A higher urinary sodium excretion has been reported in patients with autosomal dominant kidney disease (ADPKD) receiving TLV therapy, and might explain the tissue sodium mobilisation that we were able to illustrate in our patient.^23^

Another explanation could be the observed changes in the *renin-angiotensin-aldosterone* (RAA) system: aldosterone levels dropped substantially, whereas renin levels fell only temporarily during TVL administration. The aldosterone pathway plays a crucial role in PLE pathogenesis, as blocking it by mineralocorticoid effects of budesonide has the potential to reduce PLE symptoms in some patients.^19^ Additionally, recent imaging studies suggest lymphatic vessel abnormalities to be associated with the development of PLE and, interestingly, the aldosterone pathway regulates their permeability.^24^

Previous investigations reported tissue sodium storage in patients suffering from hyperaldosteronism, which was reversed following specific treatment.^11,25^ The same aldosterone effect might have caused the pronounced tissue sodium accumulation we observed in our Fontan patient. The effect of TVL on RAAS and particularly aldosterone, might be multifactorial especially in combination with other diuretics (e.g. spironolactone).^26,27^ The decreased aldosterone level in our patient might represent a favourable TLV mechanism. The current standard treatment of volume restriction and diuretics may even exacerbate this condition by further enhancing RAAS and especially the aldosterone pathway.^27,28^

We believe that our results support the rationale of a TLV trial in univentricular patients with hyponatraemia and volume overload due to PLE. Due to the clinical improvements in our patient, we continued TLV therapy beyond this described study period. Importantly, long-term administration of high doses of TLV seem to be safe, as shown in multicentre trials of ADPKD patients receiving TLV treatment for up to 11 years.^29,30^

As our report is limited, prospective and controlled studies are needed to clarify TLV effects in Fontan patients with PLE and hyponatraemia, hypoproteinaemia, and treatment resistance, as our case illustrates.

## Conclusion

^23^Na-MRI and BMC delivered novel insights into the water and sodium homeostasis of a Fontan patients with PLE treated with TLV. TLV lowered tissue Na^+^ overload and improved clinical wellbeing.

## References

[1] Fontan F Baudet E. Surgical repair of tricuspid atresia. Thorax 1971; 26: 240–248. 5089489 10.1136/thx.26.3.240 PMC1019078

[2] Gewillig M Brown SC. The Fontan circulation after 45 years: update in physiology. Heart 2016; 102: 1081–1086. 27220691 10.1136/heartjnl-2015-307467 PMC4941188

[3] Gewillig M Brown SC Eyskens B, et al. The Fontan circulation: who controls cardiac output? Interact Cardiovasc Thorac Surg 2010; 10: 428–433. 19995891 10.1510/icvts.2009.218594

[4] Rychik J. The relentless effects of the Fontan paradox. Semin Thorac Cardiovasc Surg Pediatr Card Surg Annu 2016; 19: 37–43. 27060041 10.1053/j.pcsu.2015.11.006

[5] Earing MG Cetta F Driscoll DJ, et al. Long-term results of the Fontan operation for double-inlet left ventricle. Am J Cardiol 2005; 96: 291–298. 16018859 10.1016/j.amjcard.2005.03.061

[6] John AS Johnson JA Khan M, et al. Clinical outcomes and improved survival in patients with protein-losing enteropathy after the Fontan operation. J Am Coll Cardiol 2014; 64: 54–62. 24998129 10.1016/j.jacc.2014.04.025

[7] Khairy P Fernandes SM Mayer JE Jr, et al. Long-term survival, modes of death, and predictors of mortality in patients with Fontan surgery. Circulation 2008; 117: 85–92. 18071068 10.1161/CIRCULATIONAHA.107.738559

[8] Itkin M Piccoli DA Nadolski G, et al. Protein-losing enteropathy in patients with congenital heart disease. J Am Coll Cardiol 2017; 69: 2929–2937. 28619193 10.1016/j.jacc.2017.04.023

[9] Pose A Almenar L Gavira JJ, et al. Benefit of tolvaptan in the management of hyponatraemia in patients with diuretic-refractory congestive heart failure: the SEMI-SEC project. ESC Heart Fail 2017; 4: 130–137. 28451449 10.1002/ehf2.12124 PMC5396041

[10] Takagi K Sato N Ishihara S, et al. Effects of tolvaptan on urine output in hospitalized heart failure patients with hypoalbuminemia or proteinuria. Heart Vessels 2018; 33: 413–420. 29063302 10.1007/s00380-017-1066-4 PMC5861179

[11] Kopp C Linz P Wachsmuth L, et al. ^23^Na magnetic resonance imaging of tissue sodium. Hypertension 2012; 59: 167–172. 22146510 10.1161/HYPERTENSIONAHA.111.183517

[12] Madelin G Regatte RR. Biomedical applications of sodium MRI in vivo. J Magn Reson Imaging 2013; 38: 511–529. 23722972 10.1002/jmri.24168 PMC3759542

[13] Hammon M Grossmann S Linz P, et al. ^23^Na magnetic resonance imaging of the lower leg of acute heart failure patients during diuretic treatment. PLoS One 2015; 10: e0141336. 26501774 10.1371/journal.pone.0141336 PMC4621023

[14] Kopp C Linz P Dahlmann A, et al. ^23^Na magnetic resonance imaging-determined tissue sodium in healthy subjects and hypertensive patients. Hypertension 2013; 61: 635–640. 23339169 10.1161/HYPERTENSIONAHA.111.00566

[15] Rychik J Atz AM Celermajer DS, et al. Evaluation and management of the child and adult with Fontan circulation: a scientific statement from the American Heart Association. Circulation. Epub ahead of print 1 July 2019. DOI: 10.1161/cir.0000000000000696. 31256636

[16] Kopp C Linz P Maier C, et al. Elevated tissue sodium deposition in patients with type 2 diabetes on hemodialysis detected by ^23^Na magnetic resonance imaging. Kidney Int 2018; 93: 1191–1197. 29455909 10.1016/j.kint.2017.11.021

[17] Chamney PW Wabel P Moissl UM, et al. A whole-body model to distinguish excess fluid from the hydration of major body tissues. Am J Clin Nutr 2007; 85: 80–89. 17209181 10.1093/ajcn/85.1.80

[18] Hauptman PJ Burnett J Gheorghiade M, et al. Clinical course of patients with hyponatremia and decompensated systolic heart failure and the effect of vasopressin receptor antagonism with tolvaptan. J Card Fail 2013; 19: 390–397. 23743487 10.1016/j.cardfail.2013.04.001

[19] Rychik J Dodds KM Goldberg D, et al. Protein losing enteropathy after Fontan operation: glimpses of clarity through the lifting fog. World J Pediatr Congenit Heart Surg 2020; 11: 92–96. 31835975 10.1177/2150135119890555

[20] Konstam MA Gheorghiade M Burnett JC Jr, et al. Effects of oral tolvaptan in patients hospitalized for worsening heart failure: the EVEREST outcome trial. JAMA 2007; 297: 1319–1331. 17384437 10.1001/jama.297.12.1319

[21] Gimenez I Forbush B. Short-term stimulation of the renal Na-K-Cl cotransporter (NKCC2) by vasopressin involves phosphorylation and membrane translocation of the protein. J Biol Chem 2003; 278: 26946–26951. 12732642 10.1074/jbc.M303435200

[22] Himmerkus N Plain A Marques RD, et al. AVP dynamically increases paracellular Na^+^ permeability and transcellular NaCl transport in the medullary thick ascending limb of Henle’s loop. Pflugers Arch 2017; 469: 149–158. 27924355 10.1007/s00424-016-1915-5

[23] Minami S Hamano T Iwatani H, et al. Tolvaptan promotes urinary excretion of sodium and urea: a retrospective cohort study. Clin Exp Nephrol 2018; 22: 550–561. 29052786 10.1007/s10157-017-1475-9

[24] Mohanakumar S Telinius N Kelly B, et al. Morphology and function of the lymphatic vasculature in patients with a Fontan circulation. Circ Cardiovasc Imaging 2019; 12: e008074. 30943769 10.1161/CIRCIMAGING.118.008074

[25] Christa M Weng AM Geier B, et al. Increased myocardial sodium signal intensity in Conn’s syndrome detected by ^23^Na magnetic resonance imaging. Eur Heart J Cardiovasc Imaging 2019; 20: 263–270. 30307545 10.1093/ehjci/jey134 PMC6383057

[26] Pitt B Gheorghiade M. Vasopressin V1 receptor-mediated aldosterone production as a result of selective V2 receptor antagonism: a potential explanation for the failure of tolvaptan to reduce cardiovascular outcomes in the EVEREST trial. Eur J Heart Fail 2011; 13: 1261–1263. 22102625 10.1093/eurjhf/hfr150

[27] Kida K. New insights into tolvaptan treatment and the renin-angiotensin-aldosterone system. Int Heart J 2017; 58: 307–309. 28539577 10.1536/ihj.17-177

[28] Jujo K Saito K Ishida I, et al. Randomized pilot trial comparing tolvaptan with furosemide on renal and neurohumoral effects in acute heart failure. ESC Heart Fail 2016; 3: 177–188. 27818782 10.1002/ehf2.12088 PMC5071712

[29] Torres VE Chapman AB Devuyst O, et al. Multicenter study of long-term safety of tolvaptan in later-stage autosomal dominant polycystic kidney disease. Clin J Am Soc Nephrol 2020; 16: 48–58. 33376102 10.2215/CJN.10250620 PMC7792652

[30] Edwards ME Chebib FT Irazabal MV, et al. Long-term administration of tolvaptan in autosomal dominant polycystic kidney disease. Clin J Am Soc Nephrol 2018; 13: 1153–1161. 30026287 10.2215/CJN.01520218 PMC6086720

